# Insect-bacteria parallel evolution in multiple-co-obligate-aphid association: a case in Lachninae (Hemiptera: Aphididae)

**DOI:** 10.1038/s41598-017-10761-9

**Published:** 2017-08-31

**Authors:** Rui Chen, Zhe Wang, Jing Chen, Li-Yun Jiang, Ge-Xia Qiao

**Affiliations:** 1 Key Laboratory of Zoological Systematics and Evolution, Institute of Zoology, Chinese Academy of Sciences, Beijing, 100101 China; 2 Institute of Plant Protection, Liaoning Academy of Agricultural Sciences, Shenyang, 110161 China; 3 College of Life Sciences, University of Chinese Academy of Sciences, Beijing, 100049 China

## Abstract

Parallel phylogenies between aphid and its obligate symbiont *Buchnera* are hot topics which always focused on aphid lower taxonomic levels. Symbionts in the subfamily Lachninae are special. *Buchnera* in many lachnine species has undergone functional and genome size reduction that was replaced by other co-obligate symbionts. In this study, we constructed the phylogenetic relationships of Lachninae with a combined dataset of five genes sequenced from *Buchnera* to estimate the effects of a dual symbiotic system in the aphid-*Buchnera* cospeciation association. The phylogeny of *Buchnera* in Lachninae was well-resolved in the combined dataset. Each of the genera formed strongly supported monophyletic groups, with the exception of the genus *Cinara*. The phylogeny based on sequences from *Buchnera* was divided into five tribes according to the clades of the Lachninae hosts tree, with the phylogenies of *Buchnera* and Lachninae being generally congruent. These results first provided evidence of parallel evolution at the aphid subfamily level comprehensively and supported the view that topological congruence between the phylogenies of *Buchnera* and Lachninae would not be interfered with the other co-obligate symbionts, such as *Sarretia*, in aphid-entosymbiont association. These results also provided new insight in understanding host-plant coevolution in lachnine lineages.

## Introduction

Symbiosis reflects the most prominent aspect of biological complexity affecting the ecological and evolutionary diversification of many eukaryotic groups^1^. Endosymbionts play important roles in the evolution of interacting partners involving long-term cospeciation with their hosts^1^. As one of the most representative insect groups with mutualistic associations with endosymbionts, aphids show a highly diverse assemblage of heritable bacteria^2^. Aphids harbor an obligate symbiont, *Buchnera aphidicola*, as well as many species of facultative symbionts, including *Serratia symbiotica, Regiella insecticola*, and *Hamiltonella defensa* ^3^. These symbionts have a close association with their aphid hosts. For example, *Buchnera* supplies essential nutrients to its hosts for normal development and is the obligate symbiont in nearly all aphid species^4–7^. Compared with *Buchnera*, facultative symbionts are not required for host survival and/or reproduction and are distributed in different aphid hosts^8–10^.

As an obligate symbiont, *Buchnera* underwent a rapid genome erosion early in its evolutionary history with aphids^11, 12^, and the ancient association in the aphid-*Buchnera* symbiosis dates back to 100–250 million years ago. Since then, *Buchnera* has evolved in synchrony with aphid hosts. Previous studies that focused on lower taxonomic levels (e.g., closely related species or intraspecific lineages) usually supported parallel phylogenies and cospeciation in aphid-*Buchnera* associations^13–20^. Considering that respective life cycles correspond to different aphid lineages, the evolutionary rates of the genomes of *Buchnera* in different lineages are likely to be variable^21, 22^. Different evolutionary pressures may lead to phylogenetic incongruences across long evolutionary time scales. Thus, phylogenetic incongruence may be observed at higher taxonomic levels, while phylogenetic congruence is always observed at lower levels^19, 20^. However, few studies have involved aphid-*Buchnera* phylogenetic relationships at higher taxonomic levels.

Lachninae (Insecta: Hemiptera: Aphididae) have complex yet common relationships with endosymbionts. The evidence shows that the evolutionary rates accelerated quicker in *Buchnera* from lachnine species than *Buchnera* from species of Aphidinae and Eriosomatinae^23^. The genome size of *Buchnera* varies in different lachnine lineages as well^24^. For example, the genome of *Buchnera* of one lachnine species, *Cinara cedri*, is the smallest one of all known genomes in the genus^25^. In another lachnine species, *Tuberolachnus salignus*, a convergence in functional and genome size reduction has also been found^26^. *Buchnera* in many lachnines have lost some metabolic functions, which were replaced by the other co-obligate symbiont^27, 28^. This indicates that the mutualistic relationship between *Buchnera* and its aphid host may be occasionally supplemented or supplanted by other symbionts^20, 24, 25, 27, 28^. Moreover, some lachnine species produce dwarf males that may lack *Buchnera* ^29^. Thus, if some lost or replaced events occur, the phylogenetic relationships between *Buchnera* and aphid hosts may be affected.

Lachnine aphids feed on Coniferae and some broad-leaved plants with complicated host associations. The debate about ancestral feeding conditions of the Lachninae was a subject of great interest for many studies^30–33^. Using mitochondrial and nuclear genes, Chen *et al*.^34^ first suggested the common ancestor of Lachninae fed on the woody part of an angiosperm host in the mid-Cretaceous and then switched to conifer hosts^34^. According to phylogenetic analyses, this study provided a stable, cladistic, five-tribe classification of the subfamily: Lachnini, Stomaphidini, Tramini, Tuberolachnini and Eulachnini^34^.

In this study, we used sequences of five genes from *Buchnera* (*groEL, trpB, dnaB, ilvD* and *16S rRNA*) sampled from 52 aphid species spanning 14 genera in the subfamily Lachninae to reconstruct phylogenetic relationships. We combined the resulting topologies to the reported phylogenetic tree of Lachninae, and (1) examined the monophyly of each tribe; (2) evaluated phylogenetic congruence between *Buchnera* and Lachninae species; (3) estimated the effects of two co-obligate endosymbionts in aphid-*Buchnera* cospeciation association; and (4) explored the evolutionary relationships among lachnine aphids, *Buchnera*, and host plants.

## Results

### Phylogenetic analyses of single-gene

For each gene, the analyses yielded similar results from the ML, MP, and BI analyses (Figures S1–S5). Strains of *Buchnera* from different samples of the same species were clustered in the same clades. All topologies derived from the single-gene datasets placed most aphid species into clades corresponding to genus level except *Cinara*, *Lachnus*, and *Longistigma*. The genus *Cinara* did not form a monophyletic group in any of the single-gene analyses. The genera *Longistigma* and *Lachnus* were resolved as monophyletic in all analyses except in the *16S* and *groEL* single-gene analyses. For higher taxonomic levels, the results were less conclusive, and node support values were weaker. However, the two tribes Tuberolachnini and Stomaphidini were recovered as monophyletic across different analyses with strong support. Tramini formed a monophyletic clade in the analyses of *16S*, *groEL* and *ilvD*. Eulachnini and Lachnini were recovered as monophyletic only in analyses of the *groEL* and *dnaB* datasets.

### Phylogenetic analyses of concatenated dataset

**Figure 1:**
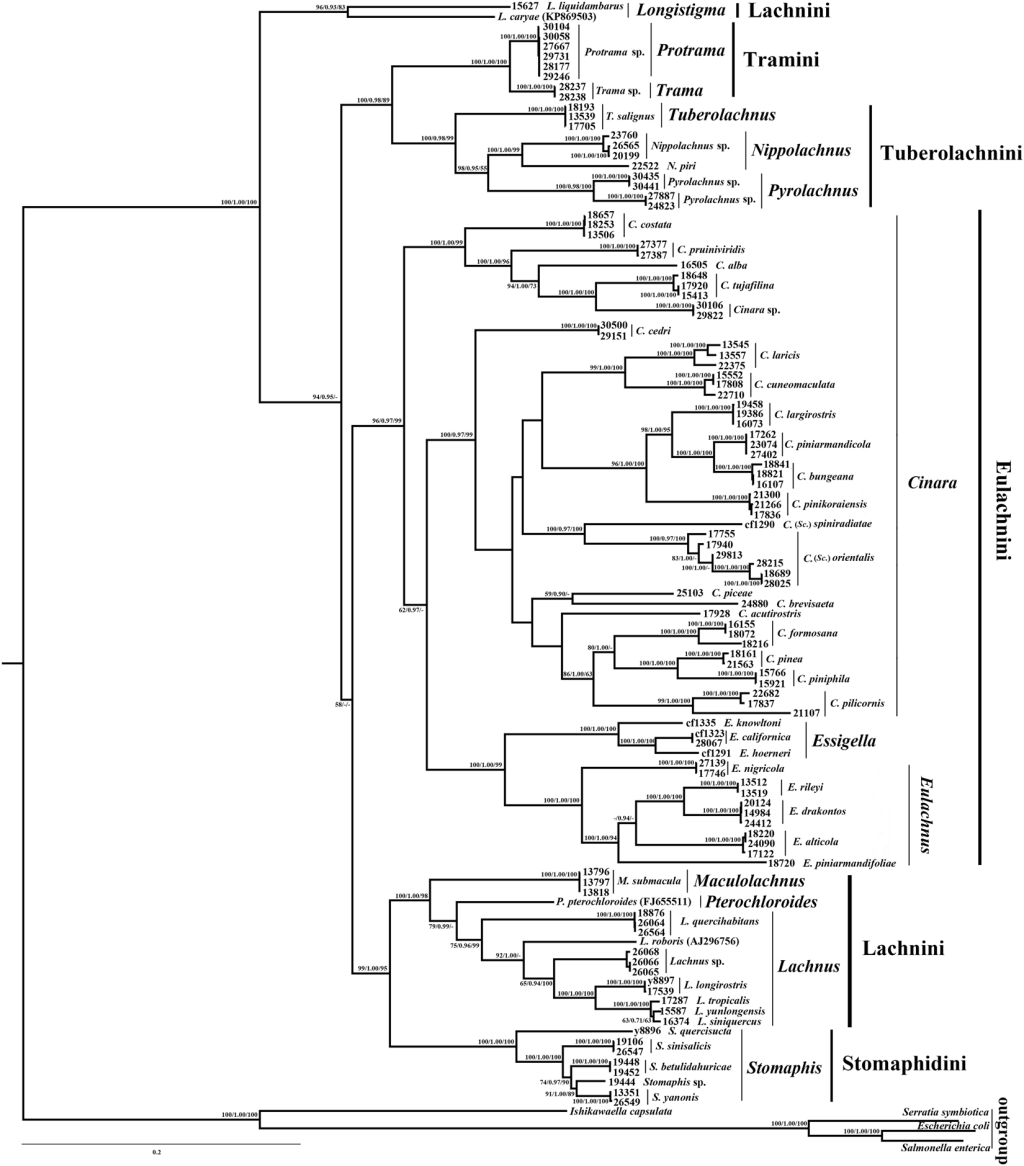
ML phylogram of *Buchnera* based on the combined dataset. The sequences of *Buchnera* species are represented by the names of their host species. The outgroup strains are designated by the names of the bacteria. The three numbers near nodes refer to ML bootstrap support, Bayesian posterior probability, and MP bootstrap support, respectively.

Maximum-likelihood, maximum-parsimony and Bayesian methods resulted in similar topologies. Here, we use the consensus tree from the combined five-gene ML analysis to summarize the results (Fig. 1). The five-gene combined analyses provided a well-resolved phylogeny. For those genera with more than one species in the analysis, average node support was also very strong (96/0.98/97, ML/MP/BI), although the genus *Cinara* was recovered as paraphyletic. The *Buchnera* tree was also divided into 5 tribes, reflecting the clades of Lachninae host phylogeny of Chen *et al*.^34^. All the tribes formed strongly supported monophyletic groups except Lachnini. Eulachnini consisted of the sister groups *Cinara* and *Essigella* + *Eulachnus*, both with strong support. Stomaphidini included all representatives of *Stomaphis*. Lachnini was divided into two groups: the genus *Longistigma* formed a monophyletic clade placed at the root of tree, and the remaining genera of *Lachnus*, *Maculolachnus*, *Pterochloroides* formed the sister-group of Stomaphidini. Tuberolachnini (*Tuberolachnus* + *Nippolachnus* + *Pyrolachnus*) and the Tramini (*Protrama* + *Trama*) were resolved as sister groups.

### Parasite-host cophylogeny analyses

**Figure 2:**
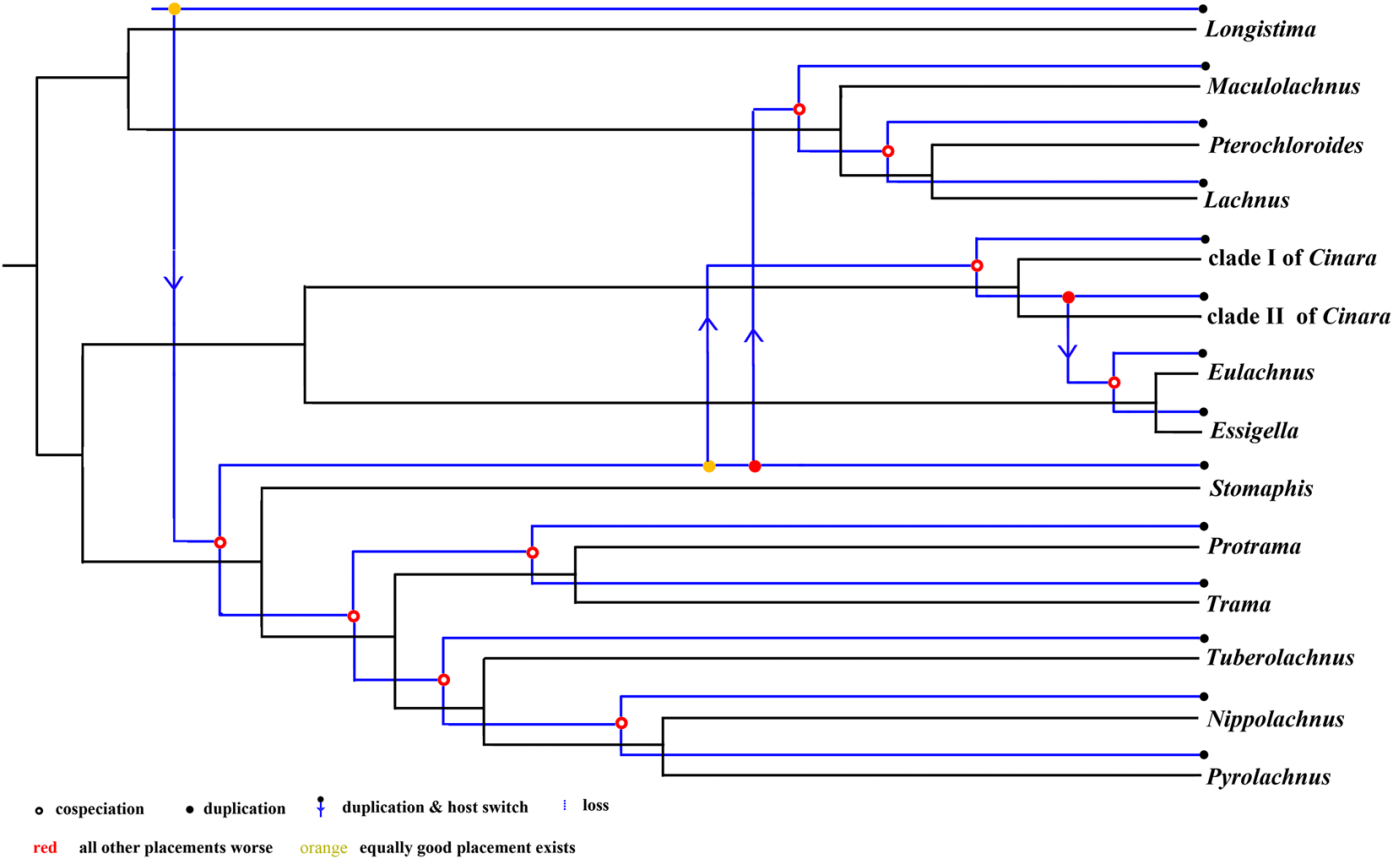
Cophylogeny of aphid and *Buchnera* from Jane at the generic level, with the reconciled trees based on the molecular-based aphid tree and the combined genes *Buchnera* tree. Blue and black lines indicate the phylogenies of the *Buchnera* and aphids, respectively. Hollow red circles indicate cospeciation events; solid red and yellow circles indicate duplications; arrows indicate host switch events; dotted lines indicate loss events.

**Figure 3:**
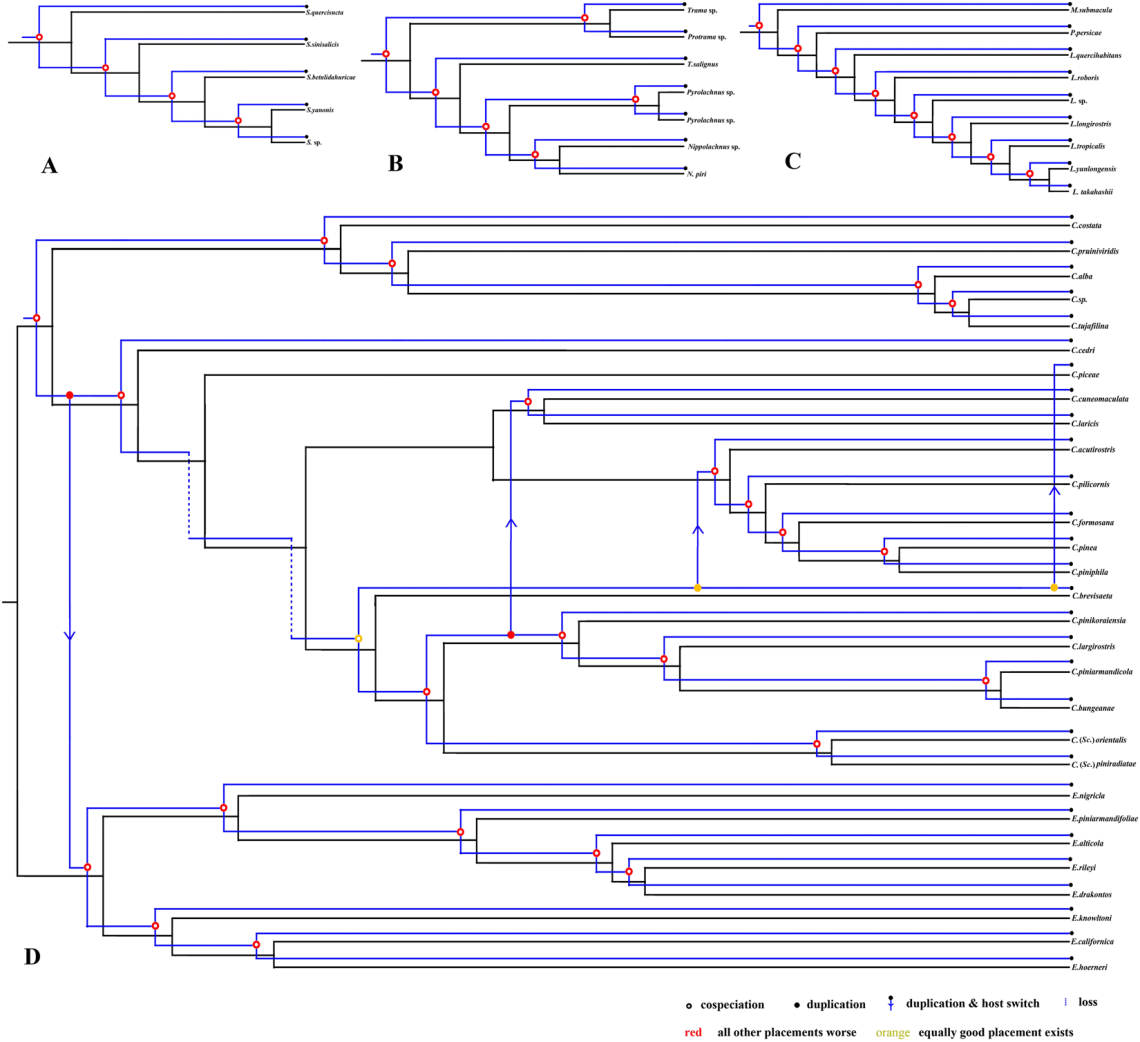
Cophylogeny of aphid and *Buchnera* from Jane at the tribe level with the reconciled trees based on the molecular-based aphid tree and the combined genes *Buchnera* tree: (**A**) Stomaphidini; (**B**) Tramini + Tuberolachnini; (**C**) Lachnini; (**D**) Eulachini. Blue and black lines indicate the phylogenies of the *Buchnera* and aphids, respectively. Hollow red circles indicate cospeciation events; solid red and yellow circles indicate duplications; arrows indicate host switch events; dotted lines indicate loss events.

To identify the relationship between *Buchnera* and Lachninae aphid hosts, we performed one reduced analysis between genera and an analysis of each tribe of Lachninae in addition to the full dataset. The cophylogeny maps were built in Jane 4.0 (Figs 2 and 3). The parameters of the coevolutionary events and ParaFit are presented in Tables 1 and 2, respectively. Analyses with ParaFit detected a significant global congruence (P = 0.001 < 0.02) between *Buchnera* and Lachninae hosts (34/50 significant links). The signal of global congruence was significant at the generic and tribal levels (Table 2). Four duplications and host switch events were detected between *Cinara* and *Essigella* + *Eulachnus, Stomaphis* and *Cinara, Stomaphis* and Lachnini (not including *Longistima*), and *Longistima* and *Stomaphis* + *Protrama* + *Trama* + *Tuberolachnus* + *Nippolachnus* + *Pyrolachnus* at the genus level (Fig. 2). The topology of *Buchnera* was in accordance with its hosts in Lachnini, Stomaphidini, and Tramini + Tuberolachnini (Fig. 3). Note that the genus *Longistigma* was excluded from the cophylogenetic analysis at the tribal level because it formed a clade that was placed at the root of the *Buchnera* phylogeny. Four duplications and host switch events were detected in Eulachnini, with three of them occurring in *Cinara* and one of them occurring between *Cinara* and *Essigella* + *Eulachnus*.

**Table 1:** The parameters obtained from event-based cophylogenetic analysis with the programs Jane and TreeMap.

		Jane				TreeMap			
		cospeciation	duplication	duplications & host switch	loss	cospeciation	duplication	host switch	sorting events
Genera		9	0	4	0	9	4	0	13
Tribes	Eulachnini	24	0	4	2	24	0	0	15
	Lachnini	8	0	0	0	8	0	0	0
	Stomaphidini	4	0	0	0	4	0	0	0
	Tramini + Tuberolachnini	6	0	0	0	6	0	0	0
All		48	0	7	2

**Table 2:** Results of the distance-based cophylogenetic analyses with ParaFit.

		P-value for global fit	Number of significant links^*^/total
Genera		0.002	4/12
Tribes	Eulachnini	0.001	11/25
	Lachnini	0.00	4/4
	Stomaphidini	0.00	8/8
	Tramini + Tuberolachnini	0.00	6/6
All		0.001	34/50

^*^refer to individual *Buchnera* -aphid associations.

## Discussion

### Dual co-obligate symbiont association would not break the phylogenetic congruence between Lachninae and *Buchnera*

The phylogenies of *Buchnera* and Lachninae were generally congruent. These results provided new evidence of parallel evolution at the aphid subfamily level. The *Buchnera* phylogeny reflected major features of the Lachninae phylogeny, including the monophyly of Tuberolachnini, Eulachnini, Tramini and Stomaphidini as well as the sister relationship between Tuberolachnini and Tramini, with strong support. These relationships are congruent with those presented by Chen *et al*.^34^, which used mitochondrial and nuclear genes to reconstruct the phylogenetic relationships of Lachninae. In addition, two methods of cophylogeny analyses all detected significant patterns of cophylogeny between *Buchnera* and its aphid hosts. These results supported the view that topological congruence between *Buchnera* and Lachninae trees would not be interfered with the other co-obligate symbionts such as *Sarretia* in aphid-entosymbiont associations. The strong cospeciation signals detected between *Buchnera* and Lachninae species could be indicative of a specialized interaction. Although the Lachninae last common ancestor lost the riboflavin biosynthetic capability by *Buchnera* that promoted the settlement of a co-obligate secondary endosymbiont^26–28^, clearly the mutualistic relationship between *Buchnera* and its aphid host has not in fact been supplanted by other symbionts. In dual symbiont associations, *Buchnera* follows a vertical mode of transmission from mother to daughter, even though certain functional genes were lost or underwent pseudogenization in evolutionary history^26–28, 35^. The species with smaller *Buchnera* genomes, such as *Cinara cedri* and *Tuberolachnus salignus* ^25, 26^, display the same relationship with other species in all aphid and *Buchnera* trees based on the combined dataset. All analyses indicated that *Buchnera* diversified in parallel with the radiation of lachnines.

Some slight incongruence between analyses based on *Buchnera* and aphid combined datasets were observed in the tribe Lachnini and the genus *Cinara*. Lachnini and *Cinara* (*Schizolachnus*) + *Cinara* were monophyletic in all aphid trees^34^. Based on the combined datasets of *Buchnera*, Lachnini was polyphyletic, and the *Longistigma* was basal to the other genera. *Cinara* (*Schizolachnus*) was nested within *Cinara*, and *Cinara* (*Schizolachnus*) + *Cinara* form two lineages branching from basal nodes in Eulachnini. Four host switch events were detected in genera *Cinara* (Figs 2 and 3). Four host switch events were also found among genera of Lachninae (Fig. 2). If the host switches occurred at deeper levels of evolutionary divergence, the aphid–*Buchnera* phylogenies should show significant incongruence. Thus, we suggest that the slight conflict between aphid and *Buchnera* trees in Lachninae may be caused by methodological artifacts, including the inadequacy of the models of evolution or limited taxon sampling as well as the lack of adequate signal for certain nodes^13, 20, 36^.

### *Buchnera* confirmed the evolutionary relationship between Lachninae and its host plants

The congruence in the phylogenetic trees of Lachninae and *Buchnera* supports cospeciation of lachnines and their primary endosymbionts following the common ancestor of aphid-*Buchnera* association. Research dated the cospeciation of *Buchnera* and the common ancestor of aphids approximately 100–250 Ma^37, 38^, and the most recent common ancestor of Lachninae dates to approximately 95 Ma^34^. These results suggested a model of a single infection in the common ancestor of modern lachnines and then stable vertical transmission of *Buchnera* from mothers to daughters. Thus, *Buchnera* genes have the potential to be used to investigate aphid-plant evolutionary history.

Lachninae show complicated host associations with multiple host switches over evolutionary history. The ancestral feeding condition of Lachninae from angiosperm or conifer host is a controversial topic receiving much attention^30–33^. Analyses of mitochondrial and nuclear genes provide strong evidence that the Lachninae common ancestor fed on the woody part of an angiosperm host, and the subsequent radiation on conifers was a derived condition^34^. In this study, endosymbiont genes provide new insight in understanding host-plant associated evolution in lachnine lineages.

Based on the gene sequences from *Buchnera*, *Longistigma* was basal within Lachninae and formed the sister group to the rest of the Lachninae. As a typical aphid group from deciduous trees, *Longistigma* feeds on bark in broad-leaved trees such as *Juglans* and *Liquidambar* ^34, 39^. This is attributed to the recurring view that angiosperm-feeding is an ancient habit in Lachninae. Moreover, the more derived position of the conifer-feeding species of Eulachnini in the *Buchnera* tree suggest a shift from angiosperm-feeding to conifer-feeding. Feeding-site specificity in Eulachnini has been suggested as a means towards reproductive isolation and thus speciation in this tribe^34, 36, 40^. Three needle-feeding taxa, including *Cinara* (*Schizolachnus*), *Eulachnus* and *Essigella*, display two clades with a more derived position in Eulachnini. It provides new evidence that needle-feeding may be a synapomorphy and has evolved more than once in Eulachnini^34^.

The close relationship between Tuberolachnini and Tramini has been confirmed, which is consistent with previous results^33, 34^. Tuberolachnini originated in East Asia and feeds on *Pyrus* and *Eriobotrya* (Rosaceae)^34^. Tramini consists of root-feeders with strictly asexual reproduction in the Palearctic region^34^. However, the evolution of the aphid niche between the bark in Rosaceae and the root from Asteraceae is unclear. In addition, the monophyly of Stomaphidini was supported in our results with strong support. The trunk-feeding Stomaphidini show strict host specialization and are diversified from a common ancestor of aphid-*Buchnera* association.

In summary, we propose an evolutionary relationship between *Buchnera* and its host Lachninae. Our findings provide the first powerful evidence of parallel evolution at the aphid subfamily level. We believe that topological congruence between *Buchnera* and Lachninae trees would not be interfered with the other co-obligate symbionts, such as *Sarretia*, in aphid-entosymbiont associations. *Buchnera* played an important role in understanding host-plant-associated evolution in lachnine lineages.

## Methods

### Taxon Sampling and data collection

The samples included most genera recognized in Lachninae. For each genus, as many species as possible were sampled, with two or more individuals of each species included. Information about aphid samples, including locations and host plants, is listed in Table S1. All samples were preserved in 95% or 100% ethanol. Three to five individuals per sample were used as slide-mounted specimens for morphological identification. Voucher specimens were identified by their main morphological diagnostic features and were compared to previously identified specimens. All samples and voucher specimens were deposited in the National Zoological Museum of China, Institute of Zoology, Chinese Academy of Sciences, Beijing, China.

### DNA extraction and sequencing

DNA extraction was performed with the whole body of single aphids using a Qiagen DNeasy^TM^ extraction kit (Qiagen, Germany) following the manufacturer’s instructions. Five genes, *16S rRNA*, *dnaB*, *groEL*, *ilvD*, and *trpB*, were amplified in this study. Information about the primers is listed in Table 3. The PCR amplifications were performed in a 30-μl reaction volume consisting of 3.0 μl 10 × PCR buffer, 2.4 μl dNTPs (10 mM each), 20 μl dd H_2_O, 0.6 μl of each 10 μM forward and reverse primer, and 1 unit of *Taq* DNA polymerase. Every PCR included a negative control (double-distilled water instead of DNA) to detect the contamination of reagents. The PCR conditions were as follows: 95 °C for 5 min; 35 cycles consisting of denaturation at 95 °C for 1 min, annealing temperature (Table 3) for 30 sec and extension at 72 °C for 2 min; and a final extension period at 72 °C for 10 min. The PCR products of *16S rRNA* gene were purified and ligated into the plasmid vector pMD19-T (TaKaRa, Dalian, China), and at least 20 clones from each product were sequenced on an ABI 3730 automated sequencer. Both strands of the plasmids were sequenced using universal primers (M13+, M13–) with forward and reverse reads. The PCR products of the other four genes were sequenced directly. Sequences were assembled and manually verified in SeqMan in the DNAStar* software package (DNASTAR, Inc., Madison, WI, USA). Multiple alignments were conducted with ClustalX in Mega 6.0^41^ and subsequently reduced to 1380 bp (*16S*), 1187 bp (*dnaB*), 1016 bp (*groEL*), 908 bp (*ilvD*), and 467 bp (*trpB*). These sequences were uploaded to GenBank (see Table S1 for accession numbers).

**Table 3:** List of primers used in PCR reactions and sequencing in this study.

Gene	Direction	Primer Name	Sequences (5′-3′)	Anneal. Temp. (°C)	Reference
*16S rRNA*	Forward	8–30	AGAGTTTGATCATGGCTCAGATTG	65	12
	Reverse	1507–1484	TACCTTGTTACGACTTCACCCCAG		12
*dnaB*	Forward	dnaBF70	CCWHATTCWYTAGAAGCWGAACAAT	50	58
	Reverse	dnaBR1348	TCAAATCKWGACCADTGWCCRTT		58
*groEL*	Forward	groELF3080	ATGGGHRCWCARATGGT	50	58
	Reverse	groELR4231	GACGWARWGGMGMTTCCAT		58
*ilvD*	Forward	ilvD716F	GARTTWGCTGTRAACATWCCWGAACA	53	58
	Reverse	ilvD1640R	GGTAGAGYATCGGTCTCCAA		58
*trpD*	Forward	trpD104F	GMAATTAATGGGWGCWRAAGTWAT	53	58
	Reverse	trpD586R	ARCCAAGMATGTTCAGGDC		58

### Phylogenetic analyses

Phylogenetic analyses were conducted on each of the five genes individually and the combined gene dataset (*16S* + *dnaB* + *groEL* + *ilvD* + *trpB*) using maximum-likelihood (ML), maximum-parsimony (MP), and Bayesian inference (BI) methods. To estimate congruence between datasets, we performed 100 replicates of the partition homogeneity test^42^ as implemented in PAUP*4.0^43^. The results indicated that the sequence data for the five genes were congruent (P > 0.01). ML analyses were conducted in RAxML 7.2.8 using a heuristic search with the GTRCAT model and 1000 bootstrap replicates^44, 45^. MP analyses were conducted in TNT v1.1 under equal weights^46^. New technology searches consisting of 10,000 random addition sequence replicates, each employing default sectorial, ratchet, drift and tree-fusing parameters, were applied. The best trees were then resubmitted for tree bisection and reconnection (TBR) branch swapping to check for additional most parsimonious trees. Clade support was assessed with 1000 bootstrap replicates^47^. The Bayesian phylogenetic analysis was conducted in MrBayes 3.1.2^48^. Appropriate evolution models were identified by evaluating the selected parameters using the Bayesian Information Criterion (BIC) in jModelTest 0.1.1^49, 50^. In MrBayes, trees were sampled every 100 generations, and the first 25% of samples were discarded as burn-in. From the post-burn-in trees, a 50% majority-rule consensus tree was generated and posterior probabilities were calculated. The sequences of *Buchnera* species are represented by the names of their host species in the phylogenetic trees.

Sequences of *Buchnera* in Lachninae from other studies downloaded from GenBank were selected as reference sequences, and *Ishikawaella capsulata*, *Salmonella enterica*, *Escherichia coli*, and *Serratia symbiotica* were chosen as outgroups (Table S2)^51^.

### Parasite-host cophylogeny analyses

Cophylogenetic analysis methods can be classified into event-based methods and distance-based methods^52^. The aim of event-based methods are to reconcile the topologies of the host and symbiont trees by adequately mixing general kinds of coevolutionary events, such as cospeciation, host-switching, duplication, etc., and finding the best reconstructions by minimizing the global cost^53^. Distance-based methods use distance matrices rather than tree topologies to test the null hypothesis that the diversification of hosts and parasites is independent. The null hypothesis is tested by permuting a host–parasite association matrix. Each individual host–parasite association can also be tested. Two event-base methods (Jane 4.0 and TreeMap v1.0)^54, 55^ and a distance-based method (ParaFit)^56^ implemented in CopyCat^57^ were used in this study. Phylogenetic relationships among the aphid species in Lachninae used in this study came from the study of Chen *et al*.^34^.

In Jane, the following event-cost scheme was used with 100 generations and a population size of 50: Cospeciation = 0, Duplication = 1, Duplication and Host switching = 2, Loss = 1, Failure to diverge = 1. Randomizations of the tips of the trees and the parasite tree topology were run in a configuring statistical test with a sample size of 1000. In TreeMap, exact and heuristic searches were used to find the best reconstructions that attempt to maximize the number of cospeciation events and minimize the number of non-cospeciation events. One thousand random replicates were run on each reconstruction to test whether the two observed phylogenies contain more cospeciation events than randomly expected by chance. ParaFit used matrices of phylogenetic distances for both hosts and parasites. Three types of information are used to describe the situation in matrix form: a matrix of phylogenetic distances among parasites, a matrix of phylogenetic distances among hosts, and a matrix of the observed host-parasite associations. All the combined data of parasitoids and hosts were used to statistically assess the global fit between trees and the significance of the contribution of each individual link between taxa to this global congruence. Tests of significance were performed using 999 permutations.

## Electronic supplementary material

Supplementary materials
