# Supplementary material for: Insect-bacteria parallel evolution in multiple-co-obligate-aphid association: a case in Lachninae (Hemiptera: Aphididae)
Source: Sci Rep. 2017 Aug 31;7:10204. doi: 10.1038/s41598-017-10761-9 (PMC5579299; doi:10.1038/s41598-017-10761-9)
Supplement: Supplementary file 1 — Supplementary materials [file 41598_2017_10761_MOESM1_ESM.pdf]

Insect-bacteria parallel evolution in multiple-co-obligate-aphid association: a case in Lachninae (Hemiptera: Aphididae)

RUI CHEN[1,†], ZHE WANG[2,†], JING CHEN[1*], LI-YUN JIANG[1], GE-XIA QIAO[1,3*]

[1] Key Laboratory of Zoological Systematics and Evolution, Institute of Zoology, Chinese Academy of Sciences, Beijing 100101, China; [2] Institute of Plant Protection, Liaoning Academy of Agricultural Sciences, Shenyang 110161, China; [3] College of Life Sciences, University of Chinese Academy of Sciences, Beijing 100049, China

† These authors contributed equally to this work.

* Correspondences: Ge-Xia Qiao, Key Laboratory of Zoological Systematics and Evolution, Institute of Zoology, Chinese Academy of Sciences, Beijing, 100101, China. E-mail: qiaogx@ioz.ac.cn; Jing Chen, Key Laboratory of Zoological Systematics and Evolution, Institute of Zoology, Chinese Academy of Sciences, Beijing, 100101, China. E-mail: chenjing@ioz.ac.cn.

Running title: Insect-bacteria parallel evolution in Lachninae

Tables of supplementary materials

Table S1. The detailed collected information of Lachninae species and GenBank accession numbers of *Buchnera aphidicola*.

Host plant	Location	No.voucher	GB number/16S	GB number/dnaB	GB number/groEL	GB number/ilvD	GB number/trpB
Salix sp.	CHINA: Shandong: Qingdao City	13351	KP866502	—	KY808126	KY808224	—
Taxus sp.	CHINA: Tibet: Gongbo'gyamda County	13506	KP866420	KY808048	KY808127	—	KY808314
Pinus tabulaeformis	CHINA: Tibet: Bayi Town	13512	KP866466	—	KY808128	—	—
Pinus densata	CHINA: Tibet: Linzhi County	13519	KP866467	—	KY808129	—	—
Salix sp.	CHINA: Tibet: Linzhi County	13539	KP866504	KY808049	KY808130	KY808225	
Larix sp.	CHINA: Tibet: Gongbo'gyamda County	13545	KP866430	KY808050	KY808131	KY808226	KY808315
Larix chinensis	CHINA: Shaanxi : Mei County	13557	KP866431	KY808051	KY808132	KY808227	KY808316
Rosaceae	CHINA: Xinjiang: Nilka County	13796	KP866487	KY808052	KY808133	KY808228	—
Rosaceae	CHINA: Xinjiang: Nilka County	13797	KP866488	KY808053	KY808134	KY808229	—
Phaseolus vulgaris	CHINA: Xinjiang: Nilka County	13818	KP866489	KY808054	KY808135	KY808230	—
Pinus tabulaeformis	CHINA: Jilin: Changbai County	14984	KP866457	—	KY808136	KY808231	KY808317
Platycladus orientalis	CHINA: Tibet: Linzhi County	15413	KP866448	KY808055	KY808137	KY808232	—
Larix sp.	CHINA: Sichuan: Wolong Town	15552	KP866423	KY808056	KY808138	KY808233	KY808318
Cyclobalanopsis glauca	CHINA: Guizhou: Daozhen County	15587	KP866483	KY808057	KY808139	—	—
Liquidambar formosana	CHINA: Guizhou : Daozhen County	15627	KP866486	—	KY808140	KY808234	—
Pinus sylvestris	CHINA: Inner Mongolia: Jiagedaqi District	15766	KP866444	KY808058	KY808141	KY808235	KY808319
Pinus sylvestris	CHINA: Inner Mongolia: Hulun Buir	15921	KP866445	KY808059	KY808142	KY808236	KY808320
Pinus tabulaeformis	CHINA: Beijing	16073	KP866429	KY808060	—	—	KY808321
Pinus bungeana	CHINA: Beijing	16107	KP866414	KY808061	KY808143	KY808237	KY808322
Pinus tabuliformis	CHINA: Beijing	16155	KP866426	KY808062	KY808144	KY808238	KY808323
Quercus sp.	CHINA: Tibet: Chayu County	16374	KP866479	KY808063	KY808145	—	—
Picea asperata	CHINA: Tibet: Zhangmu Town	16505	KP866413	KY808064	KY808146	KY808239	KY808324
Pinus yunnanensis	CHINA: Sichuan: Yanbian County	17122	KP866455	—	KY808147	—	—
Pinus armandi	CHINA: Sichuan: Meigu County	17262	KP866437	KY808065	KY808148	—	KY808325

Castanea mollissima	CHINA: Shaanxi: Foping County	17287	KP866480	KY808066	KY808149	KY808240	—
Platycladus orientalis	CHINA: Shaanxi : Foping County	17290	KY808034	KY808067	KY808150	KY808241	—
Quercus sp.	CHINA: Liaoning: Anshan City	17539	KP866473	—	KY808151	KY808242	—
Salix sp.	CHINA: Heilongjiang: Mishan City	17705	KP866505	KY808068	KY808152	KY808243	—
Pinus sylvestris	CHINA: Heilongjiang: Fujing City	17746	KP866460	—	KY808153	KY808244	KY808326
Pinus sylvestris	CHINA: Heilongjiang: Fujing City	17755	KP866468	KY808069	KY808154	KY808245	KY808327
Larix sp.	CHINA: Heilongjiang: Yichun City	17808	KP866424	KY808070	KY808155	KY808246	KY808328
Pinaceae	CHINA: Heilongjiang: Yichun City	17836	KP866441	KY808071	KY808156	KY808247	KY808329
Picea asperata	CHINA: Heilongjiang : Yichun City	17837	KP866434	KY808072	KY808157	KY808248	KY808330
Pinus sylvestris	CHINA: Inner Mongolia: Yakeshi City	17928	KP866412	—	—	—	KY808331
Pinus sylvestris	CHINA: Inner Mongolia: Yakeshi City	17940	KY808035	KY808073	KY808158	KY808249	KY808332
Pinus massoniana	CHINA: Fujian : Wuyi Mountains	18072	KP866427	KY808074	KY808159	KY808250	KY808333
Pinus yunnanensis	CHINA: Yunnan: Kunming City	18161	KP866439	KY808075	KY808160	KY808251	KY808334
Salix sp.	CHINA: Yunnan: Chuxiong City	18193	KP866506	KY808076	KY808161	KY808252	—
Pinus yunnanensis	CHINA: Yunnan : Lijiang City	18216	KP866428	KY808077	KY808162	KY808253	KY808335
Pinus yunnanensis	CHINA: Yunnan: Lijiang City	18220	KY808036	—	KY808163	—	—
Abies sp.	CHINA: Yunnan: Lugu Lake	18253	KP866421	KY808078	KY808164	—	KY808336
Platycladus orientalis	CHINA: Yunnan: Ninglang County	18648	KP866449	KY808079	KY808165	KY808254	—
Abies sp.	CHINA: Yunnan : Shangrila County	18657	KP866422	KY808080	KY808166	—	KY808337
Pinus yunnanensis	CHINA: Deqin County	18689	KY808037	KY808081	KY808167	KY808255	KY808338
Pinus armandi	CHINA: Yunnan: Jianchuan County	18720	KP866462	—	KY808168	KY808256	KY808339
Pinus bungeana	CHINA: Beijing	18821	KP866415	KY808082	KY808169	KY808257	KY808340
Pinus bungeana	CHINA: Beijing	18841	KP866416	KY808083	KY808170	KY808258	KY808341
Fagaceae	CHINA: Guangxi: Guilin City	18876	KP866475	KY808084	KY808171	KY808259	—
Salix sp.	CHINA: Beijing	19106	KP866499	KY808085	KY808172	KY808260	—
Keteleeria fortunei	CHINA: Liaoning: Shenyang City	19386	KY808038	KY808086	—	—	KY808342
Carya cathayensis	CHINA: Beijing	19444	KP866501	KY808087	KY808173	KY808261	—
Betula sp.	CHINA: Beijing	19448	KP866496	KY808088	KY808174	KY808262	—
Betula sp.	CHINA: Beijing	19452	KP866497	KY808089	KY808175	KY808263	—
Pinus tabulaeformis	CHINA: Beijing	19458	KY808039	KY808090	—	—	KY808343
Pinus tabulaeformis	CHINA: Liaoning: Anshan City	20124	KP866458	—	KY808176	KY808264	KY808344

Pyrus pyrifolia	CHINA: Anhui: Yuexi County	20199	KP866490	KY808091	KY808177	KY808265	—
Picea asperata	CHINA: Ningxia: Jingyuan County	21107	KP866435	KY808092	KY808178	KY808266	KY808345
Pinus koraiensis	CHINA: Jilin: Longjing City	21266	KP866442	KY808093	KY808179	KY808267	KY808346
Pinus koraiensis	CHINA: Jilin: Yanbian City	21300	KP866443	KY808094	KY808180	KY808268	KY808347
Pinus tabulaeformis	CHINA: Ningxia: Guyuan City	21563	KP866440	KY808095	KY808181	KY808269	KY808348
Larix sibirica	MONGOLIA: Ulan Bator City	22375	KP866432	KY808096	KY808182	KY808270	KY808349
Eribotrya japonica	CHINA: Sichuan: Chengdu City	22522	KP866493	KY808097	KY808183	KY808271	—
Pinaceae	CHINA: Sichuan: Batang County	22682	KP866436	KY808098	KY808184	KY808272	KY808350
Larix sp.	MONGOLIA: Kente Mountain	22710	KP866425	KY808099	KY808185	KY808273	KY808351
Pinus armandi	CHINA: Gansu: Tianshui City	23074	KY808040	KY808100	KY808186	—	KY808352
Pyrus sp.	CHINA: Yunnan: Jingdong County	23760	KP866491	KY808101	KY808187	KY808274	—
Pinus yunnanensis	CHINA: Yunnan: Longling County	24090	KP866456	—	KY808188	—	—
Pinus tabulaeformis	CHINA: Beijing	24412	KP866459	—	KY808189	KY808275	KY808353
Eribotrya japonica	CHINA: Yunnan: Chenghai Lake	24823	KY80804	KY808102	—	KY808276	KY808354
Pinus armandi	CHINA: Yunnan: Kunming City	24880	KP866417	—	—	—	—
Picea asperata	CHINA: Inner Mongolia: Alashan Left City	25103	KP866433	KY808103	KY808190	KY808277	KY808355
Fagaceae	CHINA: Guangxi: Lingui County	26064	KP866476	KY808104	KY808191	KY808278	—
Fagaceae	CHINA:Guangxi: Lingui Country	26065	KP866484	KY808105	KY808192	KY808279	—
Fagaceae	CHINA:Guangxi: Lingui Country	26066	KY80804	KY808106	KY808193	KY808280	—
Fagaceae	CHINA:Guangxi: Huanjiang Country	26068	KY80805	KY808107	KY808194	KY808281	—
Salix babylonica	CHINA: Shandong: Qingdao City	26547	KP866500	KY808108	KY808195	KY808282	—
Celtis sinesis	CHINA: Shandong: Qingdao City	26549	KP866503	—	KY808196	KY808283	—
Quercus sp.	CHINA: Taiwan: Taibei City	26564	KP866477	KY808109	KY808197	KY808284	—
Pyrus sp.	CHINA: Taiwan: Taizhong County	26565	KP866492	KY808110	KY808198	KY808285	—
Pinus tabuliformis	RUSSIA: St.Petersburg City	27139	KP866461	—	KY808199	KY808286	KY808356
Picea asperata	CHINA: Inner Mongolia: Alashan Left City	27377	KP866446	KY808111	KY808200	KY808287	—
Picea asperata	CHINA: Inner Mongolia: Alashan Left City	27387	KP866447	KY808112	KY808201	KY808288	—
Pinus armandi	CHINA: Beijing	27402	KP866438	KY808113	KY808202	KY808289	KY808357
Cichorium endivia	CHINA: Inner Mongolia: Daqing Montain	27667	KY808044	—	KY808203	—	KY808358
Salicaceae	CHINA: Yunnan: Yulong Mountain	27887	KP866495	KY808114	—	KY808290	—
Pinus yunnanensis	CHINA: Yunnan: Shibao Mountain	28025	KP866471	KY808115	KY808204	KY808291	KY808359

Pinus yunnanensis	CHINA: Yunnan: Weibao Mountain	28067	KP866452	—	KY808205	KY808292	KY808360
Artemisia sp.	CHINA: Gansu: Diebu Country	28177	KP866507	—	KY808206	KY808293	—
Pinus tabuliformis	CHINA: Shannxi: Hu Country	28215	KP866470	KY808116	KY808207	KY808294	KY808361
Cichorium endivia	CHINA: Henan: Gongyi Country	28237	KP866510	KY808117	KY808208	KY808295	—
Cichorium endivia	CHINA: Henan: Gongyi Country	28238	KP866511	KY808118	KY808209	KY808296	—
Cedrus deodara	CHINA: Beijing	29151	KP866418	KY808119	KY808210	KY808297	—
Cichorium endivia	CHINA: Inner Mongolia: Daqing Montain	29246	KY808045	—	KY808211	KY808298	—
Artemisia sp.	CHINA: Hubei: Shennongjia	29731	KP866508	—	KY808212	KY808299	—
Pinus massoniana	CHINA: Chongqing City	29813	KP866469	KY808120	KY808213	KY808300	KY808362
Metasequoia glyptostroboides	CHINA: Chongqing City	29822	KP866450	KY808121	KY808214	KY808301	KY808363
Artemisia sp.	CHINA: Sichuan: Jiuzhaigou Valley	30058	KY808046	—	KY808215	KY808302	—
Apium graveolens	CHINA: Sichuan:Chengdu City	30104	KP866509	—	KY808216	KY808303	—
Metasequoia glyptostroboides	CHINA: Sichuan: Chengdu City	30106	KP866451	KY808122	KY808217	KY808304	KY808364
Eribotrya japonica	CHINA: Yunana: Kunming City	30435	KY808047	—	—	KY808305	KY808365
Eribotrya japonica	CHINA: Yunnan: Wen Country	30441	KP866494	—		KY808306	KY808366
Cedrus deodara	CHINA: Beijing	30500	KP866419	KY808123	KY808218	KY808307	—
Pinus ponderosa	USA: California: Los Angeles County	CF1290	KP866472	KY808124	—	KY808308	—
Pinus monophylla	USA: California: San Bernardino	CF1291	KP866453	—	KY808219	KY808309	KY808367
Pinus radiata	USA: California: Monterrey County	CF1323	—	—	KY808220	KY808310	KY808368
Pinus contorta	USA: California: El Dorado County	CR1335	KP866454	—	KY808221	KY808311	KY808369
Quercus liaotungensis	CHINA: Beijing	Y8896	KP866498	—	KY808222	KY808312	—
Quercus liaotungensis	CHINA: Beijing	Y8897	KP866474	KY808125	KY808223	KY808313	—

Table S2. The sequences of outgroup and their GenBank accession numbers.

	16S	*dnaB*	*groEL*	*ilvD*	*trpB*
Escherichia coli	CP018801	DQ324464	CU928160	CU928160	CU928160
Serratia symbiotica	NR_117521	AENX01000092	AENX01000136	CCES01000003	AENX01000230
Ishikawaella capsulata	AB0677723	NZ_AP010872	NZ_AP010872	NZ_AP010872	NZ_AP010872
Salmonella enterica	CP012346	AE014613	AE014613	AE014613	AE014613

Figures of supplementary materials

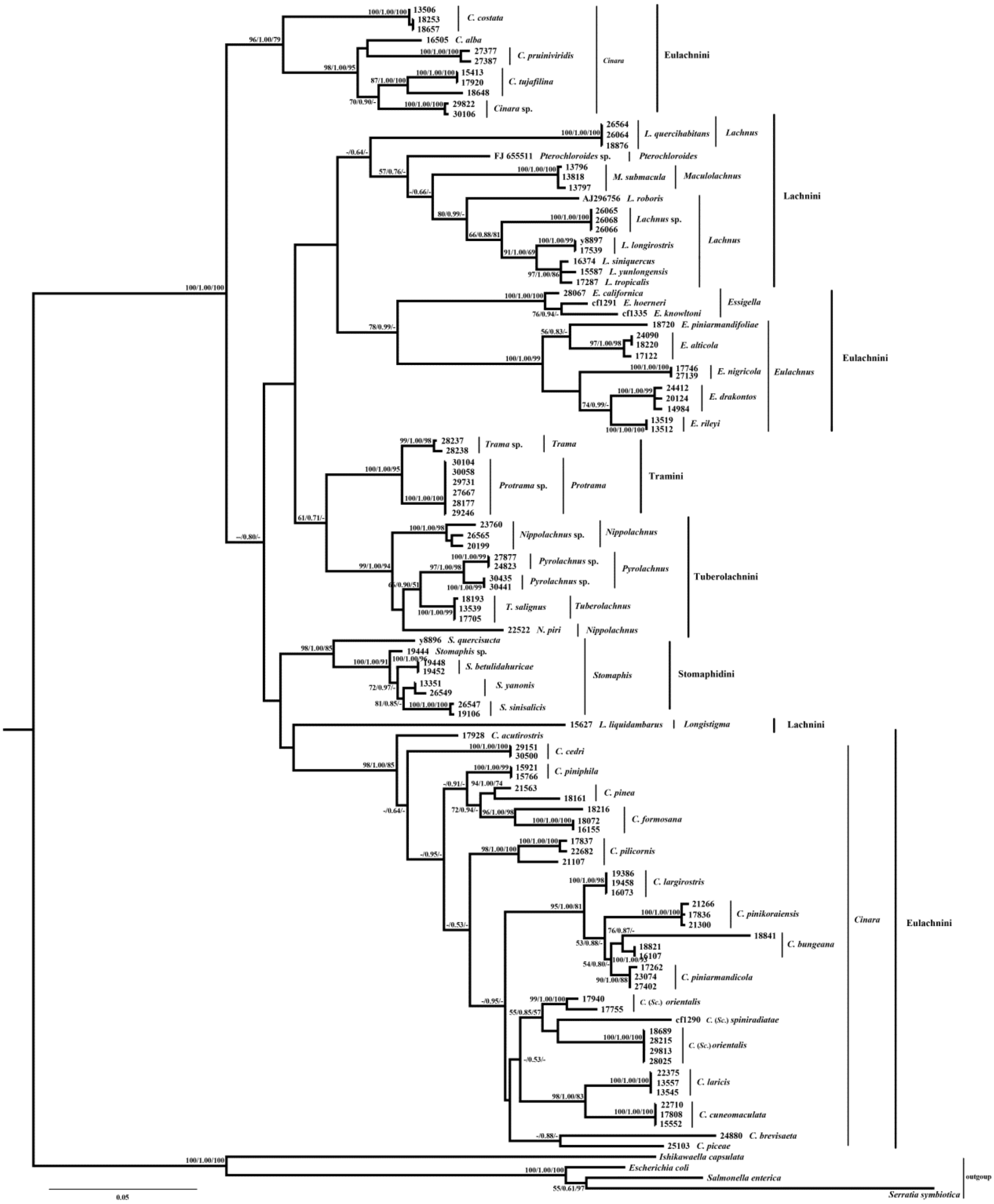

Figure S1. ML tree from the analyses of *Buchnera* in Lachninae based on the *16S rRNA* dataset. Numbers near nodes refer to ML bootstrap support, Bayesian posterior probability, and MP bootstrap support.

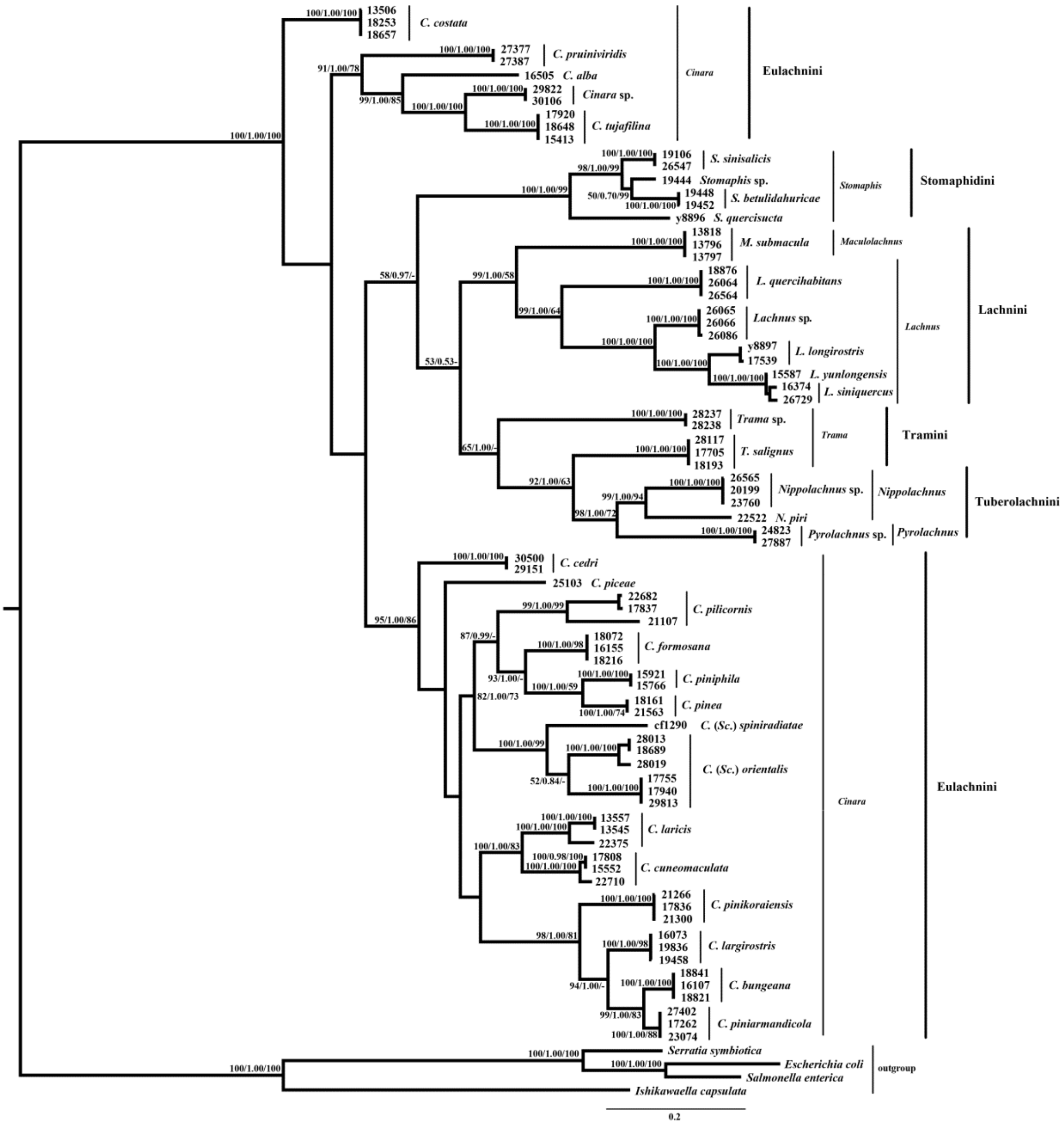

Figure S2. ML tree from the analyses of *Buchnera* in Lachninae based on the *dnaB* dataset. Numbers near nodes refer to ML bootstrap support, Bayesian posterior probability, and MP bootstrap support.

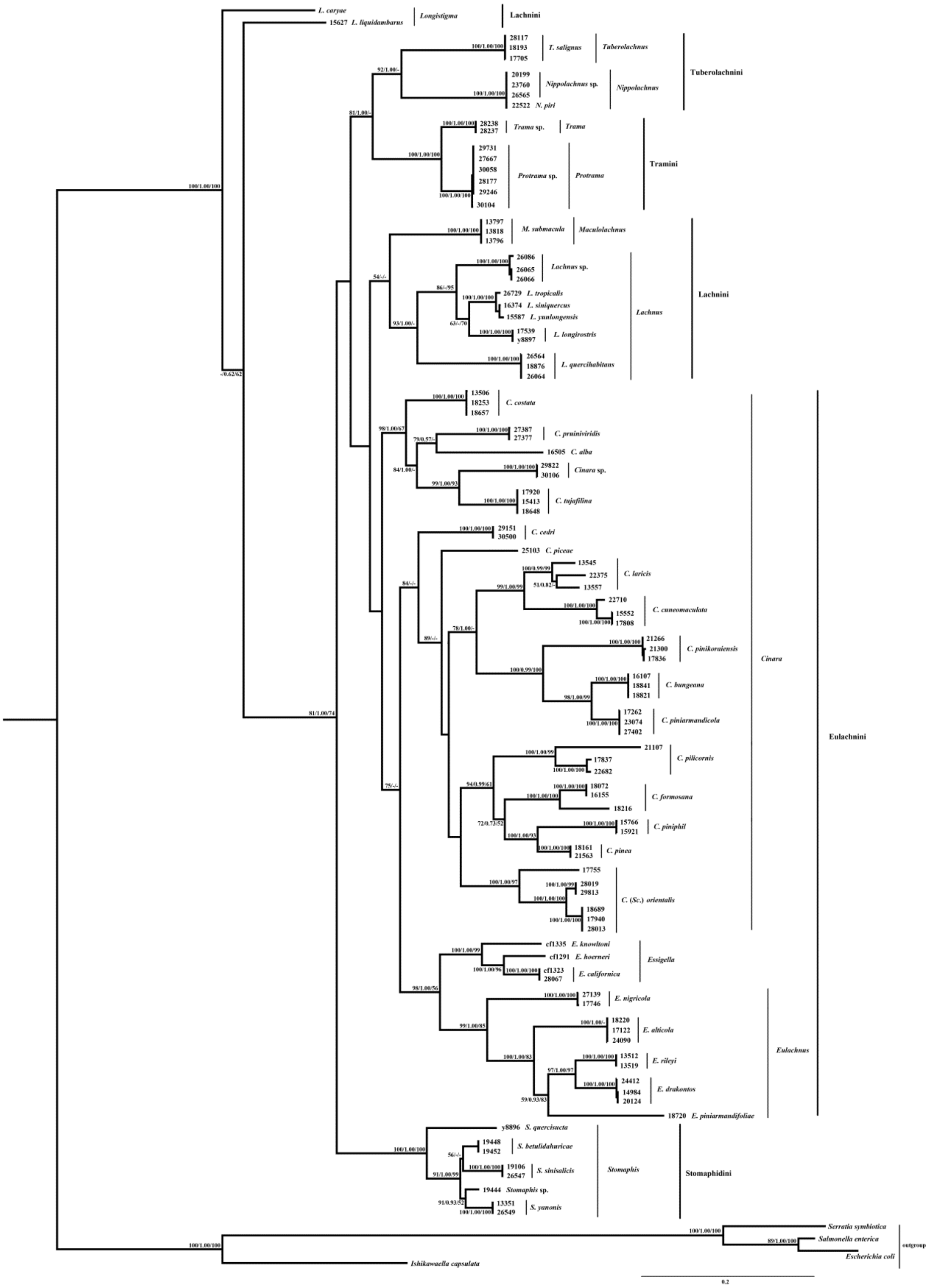

Figure S3. ML tree from the analyses of *Buchnera* in Lachninae based on the *groEL* dataset. Numbers near nodes refer to ML bootstrap support, Bayesian posterior probability, and MP bootstrap support.

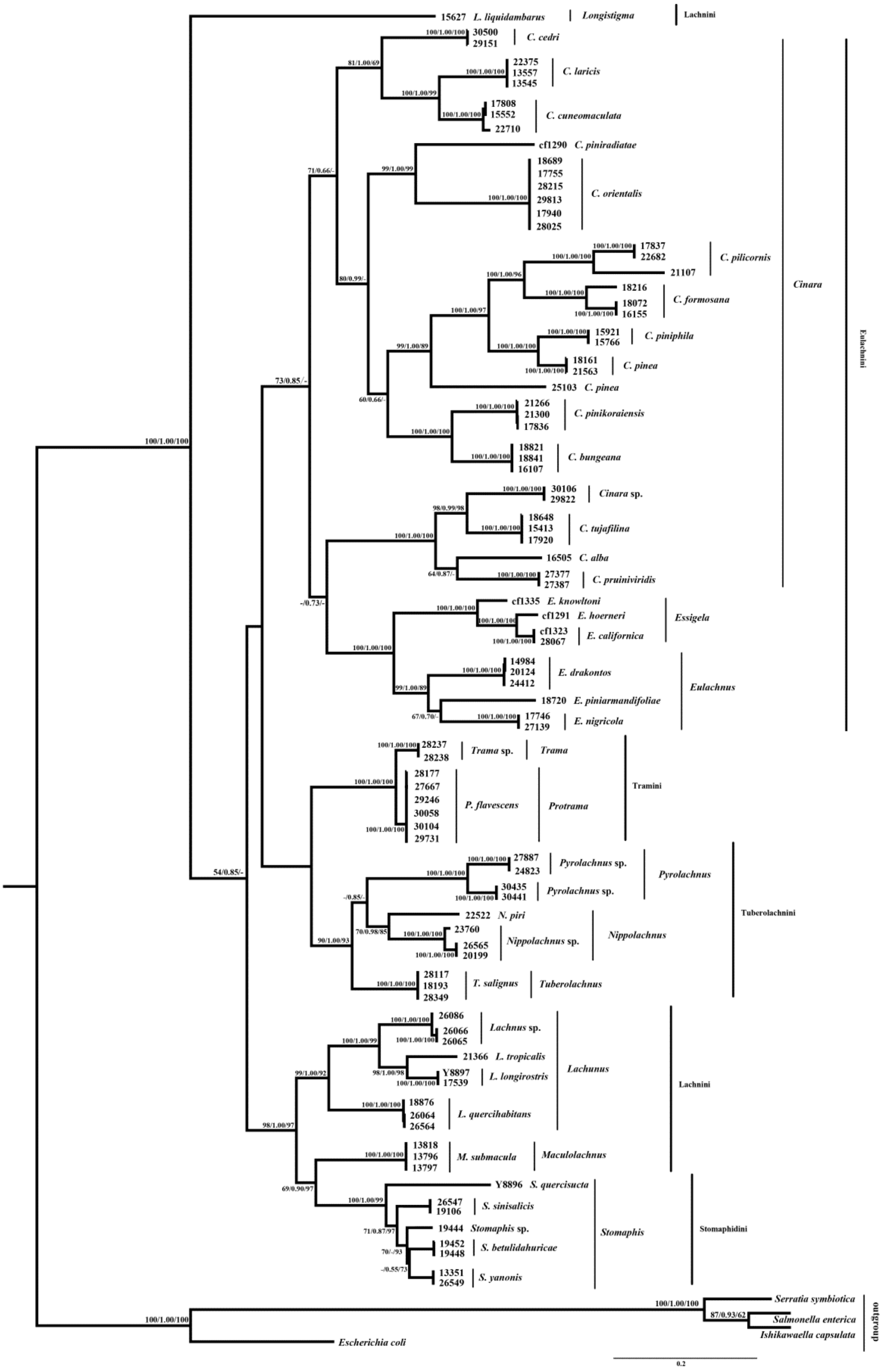

Figure S4. ML tree from the analyses of *Buchnera* in Lachninae based on the *ilvD* dataset. Numbers near nodes refer to ML bootstrap support, Bayesian posterior probability, and MP bootstrap support.

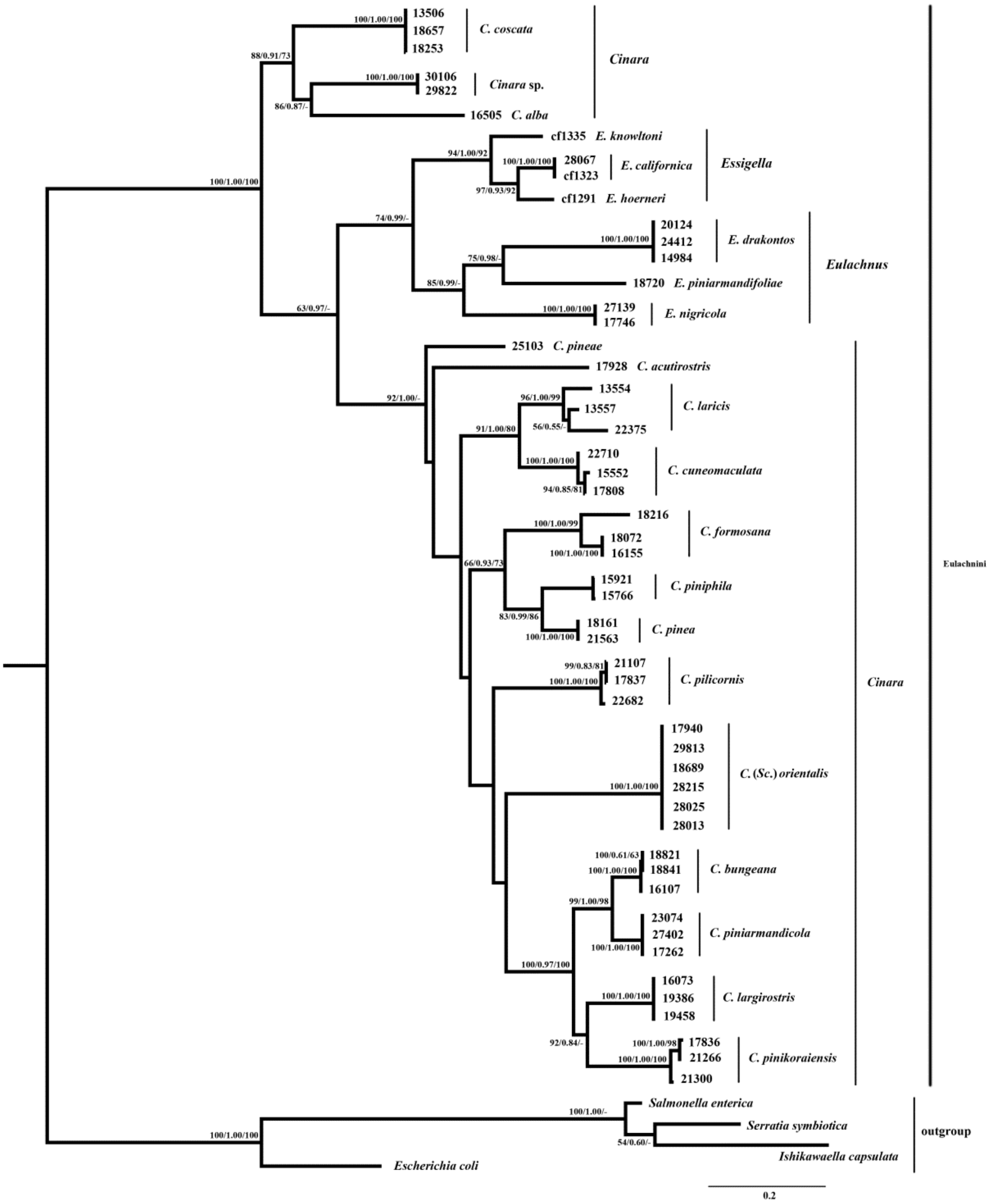

Figure S5. ML tree from the analyses of *Buchnera* in Lachninae based on the *trpB* dataset. Numbers near nodes refer to ML bootstrap support, Bayesian posterior probability, and MP bootstrap support.
